# The rubber hand illusion in hypnosis provides new insights into the sense of body ownership

**DOI:** 10.1038/s41598-020-62745-x

**Published:** 2020-03-31

**Authors:** Mirta Fiorio, Michele Modenese, Paola Cesari

**Affiliations:** Department of Neurosciences, Biomedicine and Movement Sciences, University of Verona, Verona, Italy

**Keywords:** Consciousness, Human behaviour

## Abstract

Body ownership can be experimentally investigated with the rubber hand illusion (RHI), in which watching a rubber hand stroked synchronously with one’s own hidden hand induces a feeling of ownership over the rubber hand. The aim of this study was to investigate response to the RHI in high (N = 21) and low (N = 19) hypnotizable individuals in normal waking state and in hypnosis. Response to the RHI was measured via a question on the illusory feeling of ownership and with proprioceptive drift. The Highs expressed an overall feeling of more ownership over the rubber hand in both the normal waking state and hypnosis, although both groups gave higher ownership scores after synchronous than after asynchronous stroking and the difference between conditions was similar across groups. Conversely, the proprioceptive drift appeared to be differentially modulated by hypnosis and hypnotic suggestibility: it was increased in the Highs and decreased in the Lows after hypnosis induction. These findings hint at an interplay between hypnotic suggestibility and hypnosis in modulating response to the RHI. The selective breakdown of proprioceptive drift among the Lows suggests resistance to recalibrate one’s own limb in hypnosis.

## Introduction

Sense of body ownership can be defined as experiencing the body as being part of the self. Together with the sense of agency, it is considered a fundamental component of bodily self-consciousness^1–4^. When this sense is lost, individuals may perceive their own body parts as alien, as noted in some neuropsychological (e.g., somatoparaphrenia) and neuropsychiatric (e.g., non-epileptic seizures) disorders^5,6^. Under certain circumstances, bodily self-consciousness can be compromised even in healthy individuals. An emblematic case is hypnosis. Hypnotic induction and suggestions induce in highly hypnotizable participants marked alterations in sense of agency, a lack of awareness of movement, and a sense of involuntariness^7–9^. Despite the lack of awareness, the person in hypnosis is still able to monitor and process sensory inputs. Hence, hypnosis offers a good opportunity to experimentally investigate alterations of bodily self-consciousness in healthy participants safely.

With this explorative study we wanted to investigate whether the sense of body ownership is influenced by hypnotic induction and suggestibility. To this end, we applied the rubber hand illusion (RHI)^10^, which is a frequently used paradigm to investigate sense of body ownership in a controlled experimental condition. In this paradigm, watching the synchronous stroking of a visible rubber hand with one’s own hand hidden induces a feeling of ownership over the rubber hand^10,11^. To solve the conflict between vision, proprioception, and touch, the brain recalibrates one’s own limb position toward the rubber hand with proprioceptive drift^10,11^ and combines the rubber hand with one’s own body representation, thus inducing a subjective feeling of ownership^2^. The illusion is composed mainly of two components: an illusory feeling of ownership and the proprioceptive drift. These two components seem to be influenced by specific personality traits. A previous study reported that a stronger illusory feeling of ownership is associated with higher scores on the sensory suggestibility scale^12^, which evaluates how individuals react to ongoing sensory stimulation when surreptitiously influenced by another person^13–16^ and refers to the ability to imagine a non-existent, but suggested, sensation^16^. Differently, the amount of proprioceptive drift in the RHI has been associated with hypnotic suggestibility, defined as a dispositional trait to experience suggested sensations, emotions, and thoughts during hypnotic induction^17^. This dispositional trait involves heightened attentional focus and the ability to ignore sources of interference during hypnotic induction^17^. The fact that higher hypnotic suggestibility scores were found associated with greater drift was interpreted as being due to the increased attentional capacity in highly hypnotizable individuals that, in turn, may have facilitated visuoproprioceptive integration^17^. Since Walsh and colleagues’ study was performed only in wakefulness, it should be noted that brain activation in high (Highs) and low (Lows) hypnotizable individuals is differentially modulated when they are tested in the normal wake state or in hypnosis^18–21^. Specifically, while Highs present deactivation of the anterior default mode network during hypnosis^18^ and increased activity with increasing depth of hypnosis in some prefrontal regions^20^, Lows present decreased activity also in the insula^18^, which is involved in the RHI, and especially in proprioceptive drift^22^. Based on this evidence we hypothesized that response to the RHI may vary according not only to an individual’s level of hypnotic suggestibility but also to the actual mental state. Hence, the current study is an explorative investigation into the role of normal waking state and hypnosis in influencing susceptibility to the two components of the RHI (illusory feeling and proprioceptive drift) in Highs and Lows. Our prediction is that Highs will show more proprioceptive drift than Lows in normal wakefulness, according to Walsh and colleagues^17^, and that hypnotic induction will emphasize even more the group differences due to the difference in brain activation during hypnosis in the Highs and the Lows^18–21^. Since the two components of the RHI might be differently modulated by sensory suggestibility^12^, we additionally checked for potential differences between the Highs and the Lows in this dispositional trait, which is in itself a new and informative approach. Our prediction is that higher sensory suggestibility scores will be associated with stronger illusory feeling of ownership, as found in our previous study^12^.

## Method

### Participants

In all, 55 healthy participants (25 women, mean age ± SD: 24.02 ± 3.6) volunteered to participate in the study. The number of participants we recruited is in line with other studies on the rubber hand illusion in which the same outcome measures were obtained^10–12,17,23,24^. All but two of the participants were right-handed by self-report and were recruited through advertisement at the University of Verona (Italy). Written, informed consent was obtained before starting the experiment. The study was approved by the Committee for Approval of Research on Humans (CARU), University of Verona, and conducted according to the principles expressed in the Declaration of Helsinki.

### The rubber hand illusion paradigm

For this study we used a modified version of the original rubber hand illusion (RHI) paradigm described by Botvinick and Cohen^10^. Two boxes (18 × 29 cm each) were positioned on a table. One box contained a realistic model of a right hand positioned in an anatomically plausible posture, while the other contained the participant’s right hand. The distance between the two index fingers (rubber hand and participant’s hand) was 20 cm. During stimulation, the participant’s own hand was hidden from view while the rubber hand was visible. The experimenter stroked the rubber hand and the participant’s hand by means of two paintbrushes. Small, brisk brushstrokes were applied to the dorsal surface of the middle phalanx of the index, middle, and ring fingers in a top-down direction and in randomized order between fingers. The stroking rate was approximately 1 Hz and performed for about 2 minutes. The final brushstrokes were applied to the index finger, which was the one for which participants had to give their proprioceptive judgment (see below for details). Stimulation of the two hands could be either synchronous or asynchronous. In synchronous stimulation, the experimenter tried to synchronize the movement of the two paintbrushes on the two hands as much as possible; in asynchronous stimulation, the experimenter moved the paintbrush on the rubber hand and then over the participant’s hand at a rate of approximately 1 Hz. The type of stimulation (i.e., synchronous or asynchronous) was delivered in counterbalanced order across participants.

Participants were asked to observe the rubber hand and to focus on their feelings. At the end of each condition, an explicit measure of the RHI was recorded by means of response to the statement: “I felt as if the rubber hand was my own hand”, which is the one illusion statement among those on the original questionnaire^10^ that is solely related to the illusory feeling of ownership^25^. Participants rated their agreement or disagreement with the statement on a scale from −3 (“I totally disagree”) to +3 (“I totally agree”), with 0 meaning “I do not know”. We decided not to administer the entire questionnaire in order to minimize the potential risk of participants exiting the hypnotic state due to overloading of cognitive self-assessment. In our explorative study, the asynchronous stroking condition served as control.

**Figure 1:**
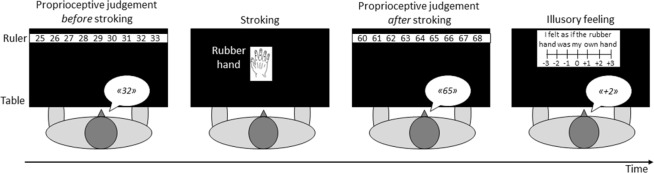
Diagram of the RHI task. Before stroking, participants were asked to state the number on a ruler indicating the position of their hidden right hand (the index finger). The rubber hand was visible during stroking. After stroking, the ruler was positioned again in front of the participants with a different onset number and they were asked to state the number indicating the position of the right index finger. The difference between the two proprioceptive judgments (final – baseline) was defined as a measure of the proprioceptive drift. Typically, there is a drift in the perceived position of one’s own hand toward the rubber hand after synchronous stroking. Finally, the participants were asked to state their degree of agreement or disagreement with the statement related solely to the illusory feeling of ownership.

To obtain an implicit measure of the illusion, we evaluated proprioceptive drift. Before starting stroking (baseline) and immediately after stroking (final), a ruler was positioned over the box in front of the participant. He/she had to report the perceived position of his/her own hand (particularly the index finger) by stating the corresponding number on the ruler without moving hands (Fig. 1). The rubber hand and the subject’s hand were out of view during proprioceptive judgment. The ruler onset and offset numbers changed each time in order to avoid response biases. The perceived position of the hand before and after stroking was measured according to a landmark, which was the edge of the box. This provided us with a precise and identical starting point of ruler placement for all subjects. The difference between the final and the baseline judgment gave a measure of the so-called “proprioceptive drift”, i.e., the proprioceptive displacement of one’s own hand towards or away from the rubber hand^11^. A difference of 0 between the final and the baseline proprioceptive judgment means that the participants perceived their own hand as being exactly in the same position before and after stroking, indicating a lack of proprioceptive displacement of their own hand towards or away from the rubber hand. Conversely, a positive difference between the final and the baseline proprioceptive judgment indicated a drift in the perception of their own hand towards the rubber hand, whereas a negative difference between the final and the baseline proprioceptive judgment indicated a drift in the perception of their own hand away from the rubber hand. Typically, a displacement of the real hand towards the rubber hand occurs after synchronous but not after asynchronous stimulation, and this difference in proprioceptive drift towards the rubber hand between the synchronous and asynchronous condition is often used as an objective measure of the illusion^11,24^.

### Procedure

**Figure 2:**
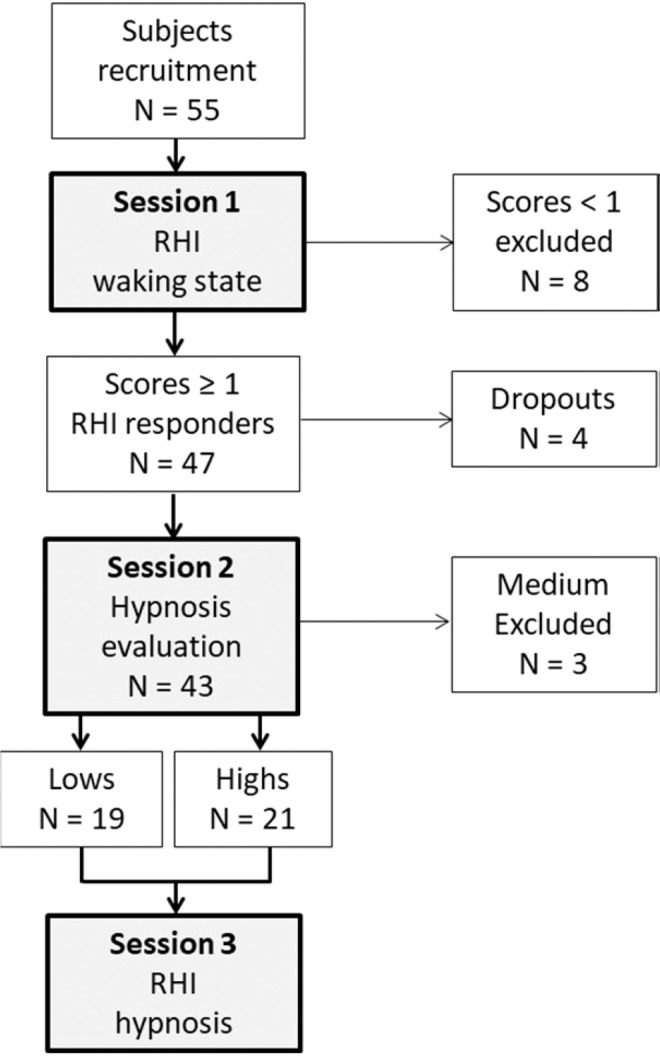
Study flow chart. During session 1, a total of 55 healthy participants were exposed to the RHI paradigm in the normal waking state and screened for the presence of the illusion (agreement scores ≥ 1 on the Likert scale). Forty-seven RHI-responders were enrolled in the study, 4 of which dropped out because of personal reasons. During session 2, 43 participants were evaluated for level of hypnotic suggestibility. Three participants had medium scores and were excluded from further testing. The 40 remaining participants were categorized as either High-responders (N = 21) or Low-responders (N = 19). During session 3, they were exposed to the RHI paradigm in the hypnotic state.

The procedure was carried out in three distinct sessions on 3 days. The first session entailed application of the RHI paradigm in the normal waking state. This session also served to select participants who experienced the illusion (RHI-responders). Namely, in order to investigate for any effect of hypnosis on the sense of body ownership, we first selected participants who experienced the illusion. We defined as RHI-responders the participants who, after synchronous stroking, agreed with the statement: “*I felt as if the artificial hand were my hand*” by giving a score between +1 and +3. In all, 47 out of 55 participants resulted RHI-responders (22 females, mean age: 23.49 ± 3.1). Four RHI-responders withdrew from the study because of personal reasons (dropouts) (Fig. 2).

In the second session, an expert hypnotist (M.M.) evaluated the RHI-responders’ level of hypnotic susceptibility. The responders were tested in groups of 10 and comfortably seated in a large, quite room in such a way that the hypnotist could approach them easily. They were asked to relax and follow the hypnotist’s words as he guided them into a hypnotic state. During this session, the hypnotist assessed the level of hypnotic susceptibility for each responder according to typical signs (e.g., head falling, eye closure, hand lowering, arm immobilization) that indicate response to hypnotic induction, as described in the Harvard Group Scale of Hypnotic Susceptibility: Form A (HGSHS:A)^26^ and according to Italian norms^27^. Participants who received scores from 1 to 4 were defined as *Lows* (N = 19, 7 females, mean age: 23.58 ± 2.68) and those who received scores from 9 to 12 were defined as *Highs* (N = 21, 11 females, mean age: 23.57 ± 3.59). Three participants with scores between 5 and 8 were defined as medium and were excluded from the analysis (Fig. 2). The distinction between Highs and Lows allowed us to investigate for differences in the degree of RHI under hypnosis in relation to level of hypnotic susceptibility.

In the third session, the remaining 40 participants were exposed to the RHI paradigm in hypnosis. This session started with induction of hypnosis by the hypnotist. More precisely, hypnosis was induced in the Highs with a post-hypnotic command by the hypnotist, whereas the Lows received an inductive trace of concentration and attentional focus on the task. After the induction phase, the participants were exposed to the RHI task as in the first session. Hypnosis lasted until the end of the RHI task (about 5 minutes), when the hypnotist re-established the normal waking state. The aim of the third session was to evaluate the amplification or inhibition of the RHI in hypnosis compared to the normal waking state (first session) and according to the individual’s level of hypnotic susceptibility.

### Sensory suggestibility scale

Suggestibility can be defined also in nonhypnotic terms. For instance, sensory suggestibility refers to an individual’s tendency to be suggestible to perceptual experiences^13–15^. Sensory suggestibility has been previously studied in the context of the rubber hand^12^. In our study we measured our participants’ sensory suggestibility as a secondary outcome to better characterize the response of the Highs and the Lows to the RHI.

To do this, we applied the sensory suggestibility scale (SSS) at the end of the experimental procedure^13–15^. The SSS score is a measure of indirect suggestibility in which the intention to influence remains concealed, with the participant unaware that her sensory suggestibility is going to be tested^16^. For this scale, the experimenter uses verbal suggestions reinforced by ad-hoc stimulation (for example, a cold metal plate placed on the skin of the forearm) to induce the participant to perceive different sensations (tactile, visual, auditory or gustatory). In this instance, the experimenter asks the participant to uncover her right forearm and to touch the inside of the forearm with a cold metal plate with the other hand. With eyes closed, the participant is then asked to remove the metal plate and to concentrate on the sensation of coldness she feels on her forearm and on the feeling of coldness and numbness radiating to the right hand. The participant is asked to report whether and how vividly she perceived the suggested sensation on a scale from 0 (no sensation) to 4 (very strong sensation). Some of the induced sensations are not physiologically plausible (in the example given here, there is no actual physiological propagation of coldness and numbness from the forearm to the hand); nonetheless, highly sensory suggestible participants typically select high scores. Control conditions are also inserted in order to effectively evoke the suggested sensation and make the test more believable. For instance, the participant is asked to close the ear with the index finger and rate the sensation of hearing an ocean-like sound. In this condition, the suggested sensation effectively occurs and scores are typically high in all individuals. For our study, we administered ten experimental conditions to induce reports of non-physiologically plausible sensations and two control conditions to induce veridical sensations^16,28^. The total score is the sum of the scores recorded for each experimental condition (range 0–40).

### Data analyses

Our primary outcome was the response to the RHI in normal waking and in hypnosis, depending on the hypnotic suggestibility level (High and Low). Preliminary analyses with the t-test for independent samples and chi-square tests were carried out to verify that the Highs and the Lows were comparable for age and gender. The illusory feeling of ownership was analyzed using non-parametric tests due to the ordinal nature of the data. We proceeded in two ways. First, we adopted a within-subjects design in which we applied the Wilcoxon signed ranks test to compare, within each group, the two stroking conditions (synchronous vs. asynchronous) separately for the two mental states (hypnosis and normal waking state). The Wilcoxon signed ranks test was also applied to compare, within each group, the two mental states (hypnosis vs. normal waking state) separately for the two conditions (synchronous and asynchronous). Second, we adopted a between-subjects design in which we applied the Mann-Whitney U test to compare the two groups (Highs vs. Lows) in each condition separately (hypnosis synchronous, hypnosis asynchronous, normal waking state synchronous, normal waking state asynchronous). Moreover, we defined an illusion index for the questionnaire rating, computed as the difference between the score obtained in the synchronous and the asynchronous condition (synchronous – asynchronous) separately for hypnosis and normal waking. The illusion index for the questionnaire rating was analyzed by means of the Wilcoxon signed-ranks test to compare the two mental states (hypnosis vs. normal waking state) within each group and by means of the Mann-Whitney U test to compare the two groups (Highs vs. Lows) in hypnosis and normal waking separately. The effect size r for non-parametric tests was computed.

Proprioceptive drift was analyzed by means of mixed model analyses of variance (ANOVA) with Condition (synchronous vs. asynchronous) and State (hypnosis vs. normal waking state) as within-subjects factors and Group (Highs vs. Lows) as between-subjects factor. Furthermore, we defined an illusion index for the proprioceptive drift, computed as the difference between the drift obtained in the synchronous and the asynchronous condition (synchronous – asynchronous), separately for hypnosis and normal waking. The illusion index for the proprioceptive drift was analyzed by means of mixed model ANOVA with State (hypnosis vs. normal waking state) as a within-subjects factor and Group (Highs vs. Lows) as a between-subjects factor. Post-hoc comparisons were performed with the t-test for paired and independent samples by applying Bonferroni correction for multiple comparisons when necessary. The effect size was calculated using η^2^ for ANOVA and Cohen’s d for the planned comparisons^29^. Finally, data on the proprioceptive drift in the two groups were analyzed against zero (with zero denoting the lack of drift) by means of the one sample t-test separately for the normal waking state and for hypnosis. Based on the way the drift was measured and computed, this analysis allowed us to determine whether an appreciable proprioceptive drift towards the rubber hand occurred in different conditions and groups.

As a secondary outcome, the two groups were compared for the level of sensory suggestibility (SSS) with the Mann-Whitney U test. Spearman’s coefficient of correlation was used to test the relation between the total SSS score and the RHI measures (i.e., illusory feeling of ownership and proprioceptive drift) obtained in each condition, state, and group.

For all analyses, p values < 0.05 were considered statistically significant. All data are expressed as mean ± standard error (SE).

## Results

Analyses of study sample demographics confirmed that the two groups were comparable for age (t(38) = −0.01, *p* = 0.994) and gender distribution (χ^2^ = 0.40, *p* = 0.527).

### Illusory feeling of ownership

**Figure 3:**
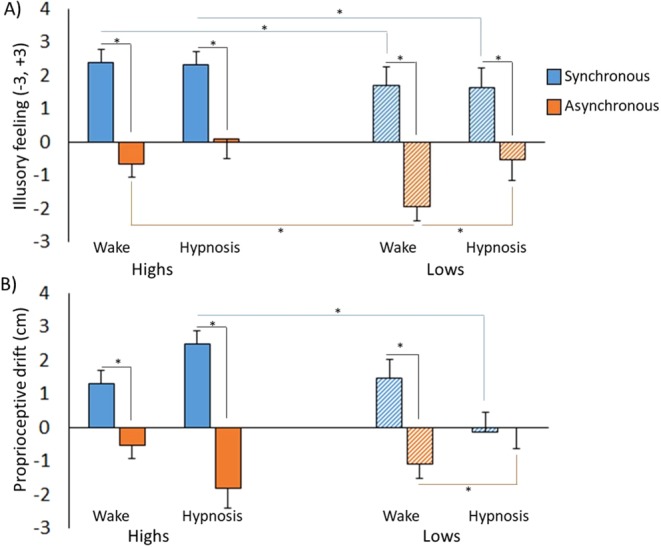
(**A**) Mean scores of the illusory feeling of ownership for the Highs (left panel, full-colored bars) and the Lows (right panel, striped-colored bars) after synchronous (blue bars) and asynchronous (orange bars) stroking in the normal waking state and in hypnosis. In both groups, the amount of illusion was higher after synchronous than asynchronous stimulation. Moreover, higher scores were recorded for the Highs than the Lows in both the normal waking state and hypnosis. (**B**) Mean scores of proprioceptive drift for the Highs (left panel, full-colored bars) and the Lows (right panel, striped-colored bars) after synchronous (blue bars) and asynchronous (orange bars) stroking in the normal waking state and hypnosis. More drift after synchronous stimulation was recorded for the Highs in hypnosis than for the Lows. Suppression of the proprioceptive drift also after asynchronous stimulation in hypnosis compared to the normal waking state was noted for the Lows. Error bars represent standard errors. *p < 0.050.

After comparing the two stroking conditions within each group for the two mental states separately (i.e., normal waking state and hypnosis), we found higher illusory scores for the Highs after synchronous (normal waking state 2.38 ± 0.18; hypnosis 2.33 ± 0.18) than after asynchronous stroking (normal waking state −0.67 ± 0.37; hypnosis 0.095 ± 0.32) in the normal waking state (Z = −3.84, *p* < 0.001, r = −0.593) and in hypnosis (Z = −3.85, *p* < 0.001, r = −0.594) (Fig. 3A). The same pattern of results was found also for the Lows, with higher illusory scores found after synchronous (normal waking state 1.68 ± 0.19; hypnosis 1.63 ± 0.19) than after asynchronous stroking (normal waking state −1.95 ± 0.39; hypnosis −0.53 ± 0.34) in the normal waking state (Z = −3.88, *p* < 0.001, r = −0.629) and in hypnosis (Z = −3.58, *p* < 0.001, r = −0.581) (Fig. 3A). This finding suggests that the illusory feeling of ownership can be evoked in different mental states independent of the level of hypnotic suggestibility.

After comparing the two mental states (hypnosis vs. normal waking state) within each group, we found a difference between hypnosis and normal waking state with regards to the asynchronous condition, selectively for the Lows (Z = −2.55, *p* = 0.011, r = −0.414), whereas no statistically significant difference between normal waking and hypnosis was found for the Highs (for both comparisons, *p* > 0.061) (Fig. 3A). This finding could indicate that under hypnosis the Lows became more uncertain in response to asynchronous stimulation.

Finally, on comparing the two groups we found higher scores for the Highs than the Lows after synchronous stroking in both the normal waking state (Z = −2.52, *p* = 0.012, r = −0.398) and hypnosis (Z = −2.57, *p* = 0.010, r = −0.406) (Fig. 3A), hinting at a tendency of the Highs to agree more than the Lows with the statement measuring the illusory feeling of ownership over the rubber hand. A between-group difference was found after asynchronous stroking in the normal waking state (Z = −2.14, *p* = 0.032, r = −0.338), with more negative values recorded for the Lows than the Highs (Fig. 3A).

**Figure 4:**
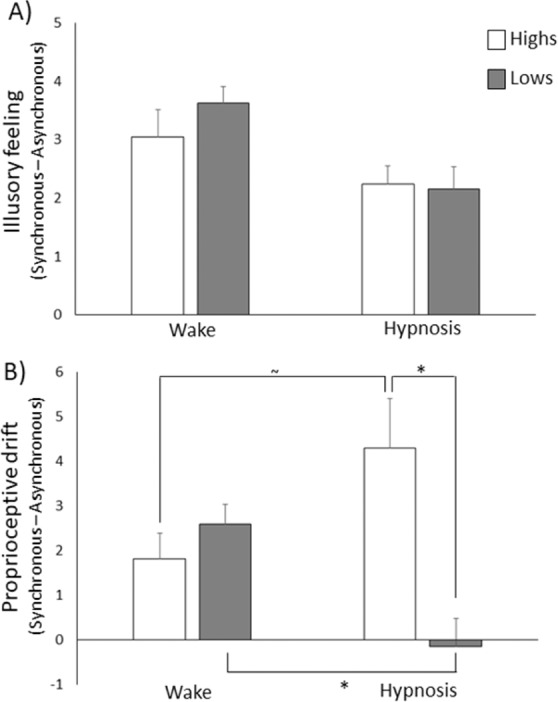
Illusion index computed as the difference between stroking conditions (synchronous – asynchronous) in the Highs (white bars) and the Lows (grey bars). (**A**) At the illusory feeling of ownership, the illusion index was overall greater in the normal waking state than in hypnosis, without differences between groups. (**B**) For the proprioceptive drift, the illusion index was decreased in the Lows and increased in the Highs after hypnotic induction. *p < 0.050; ^~^p = 0.052.

When we analyzed the illusion index (synchronous – asynchronous) by comparing the two mental states (normal waking vs. hypnosis) within each group, we found that the index was larger in the normal waking state (Highs 3.05 ± 0.47; Lows 3.63 ± 0.28) compared to hypnosis (Highs 2.24 ± 0.32; Lows 2.158 ± 0.38) for both groups (Highs Z = −2.19, *p* = 0.028, r = −0.339; Lows Z = −2.69, *p* = 0.007, r = −0.437) (Fig. 4A). No statistically significant effect emerged when we compared the two groups (*p* > 0.408).

### Proprioceptive drift

For proprioceptive drift we found a significant effect of Condition (F_(1,38)_ = 29.67, *p* < 0.001, η^2^ = 0.438) due to a higher drift toward the rubber hand after synchronous (1.27 ± 0.27 cm) than asynchronous (−0.86 ± 0.29 cm) stimulation. Moreover, the interaction Condition × Group was also significant (F_(1,38)_ = 5.52, *p* = 0.024, η^2^ = 0.127). Post-hoc comparisons revealed a stronger proprioceptive drift for the Highs (1.88 ± 0.414 cm) than the Lows (0.66 ± 0.36 cm) after synchronous stimulation (*p* = 0.027, Cohen’s d = 0.728, confidence interval, CI = [0.148 2.299]). For both groups the drift was greater after synchronous than asynchronous stimulation (for both comparisons, *p* < 0.006). The triple interaction Condition × State × Group was also significant (F_(1,38)_ = 12.58, *p* = 0.001, η^2^ = 0.249). Post-hoc comparisons showed more drift for the Highs in hypnosis than for the Lows (2.48 ± 0.6 cm vs. −0.16 ± 0.55 cm) after synchronous stimulation (*p* = 0.003, d = 1.018, CI = [0.978 4.290]) (Fig. 3B). While a significant difference between synchronous and asynchronous stimulation in both normal waking state (*p* = 0.005, d = 0.963, CI = [0.60 3.019]) and in hypnosis (*p* = 0.001, d = 1.188, CI = [1.964 6.607]) was noted for the Highs, the Lows maintained this difference between conditions only in the normal waking state (*p* < 0.001, d = 1.816, CI = [1.612 3.547]) but not in hypnosis (*p* = 0.807). Finally, a significant difference between the awake and the hypnotic state with regards to asynchronous stimulation was observed for the Lows (*p* = 0.008, d = −0.959, CI = [−1.891–0.32]): in the normal waking state the proprioceptive drift after asynchronous stimulation was present in the opposite direction with respect to the rubber hand (−1.11 ± 0.38), whereas no drift was present in hypnosis (0.0 ± 0.43).

Analysis of the illusion index (synchronous – asynchronous) revealed a significant Group effect (F_(1,38)_ = 5.52, *p* = 0.024, η^2^ = 0.127) due to a generally larger illusion index in the Highs than in the Lows, and a significant interaction State × Group (F_(1,38)_ = 12.58, *p* = 0.001, η^2^ = 0.249). Post-hoc comparisons showed that the Highs had a larger illusion index (4.29 ± 1.11) than the Lows (−0.16 ± 0.66), selectively in hypnosis (*p* = 0.002, d = 1.07, CI [1.77 7.11]). Differently, the Lows had a larger illusion index in normal waking (2.58 ± 0.46) compared to hypnosis (*p* = 0.003, d = 1.12, CI [1.08 4.4]); whereas in the Highs the comparison between the illusion index in normal waking (1.81 ± 0.58) and in hypnosis showed only a trend toward significance (*p* = 0.052, d = −0.64, CI [−4.98 0.03]) (Fig. 4B).

Analysis against zero revealed that proprioceptive drift in the Highs was significantly higher than zero (indicating a positive drift toward the rubber hand) after synchronous stroking in both the normal waking state (t_(20)_ = 3.02, *p* = 0.007, d = 0.932, CI = [0.397 2.175]) and hypnosis (t_(20)_ = 4.15, *p* < 0.001, d = 1.281, CI = [1.233 3.72]). Conversely, the drift for the Highs was significantly lower than zero after asynchronous stroking only in hypnosis (t_(20)_ = −2.48, *p* = 0.022, d = −0.765, CI = [−3.33–0.289]). In the Lows, proprioceptive drift was higher than zero after synchronous stroking (t_(18)_ = 3.68, *p* = 0.002, d = 1.194, CI = [0.633 2.314]) and lower than zero after asynchronous stroking (t_(18)_ = −2.897, *p* = 0.010, d = −0.940, CI = [−1.907–0.304]) specifically in the normal waking state, whereas no difference from zero was found in either direction in hypnosis, hinting at a lack of proprioceptive drift in this state.

### Sensory suggestibility scale

**Figure 5:**
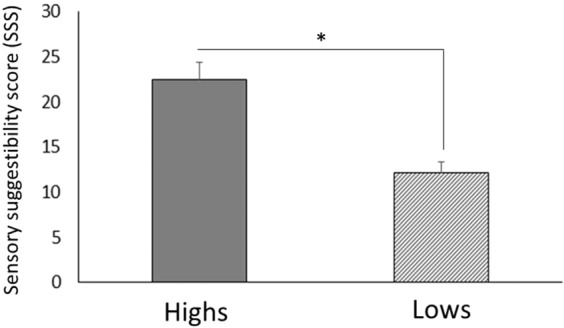
Total sensory suggestibility scale (SSS) scores for both groups. Scores were significantly higher for the Highs than the Lows. Error bars represent standard errors. *p < 0.050.

Differences in SSS scores (Z = −3.98, p < 0.001, r = −0.629) were noted between the two groups, with higher scores recorded for the Highs than the Lows (22.52 ± 1.83 vs. 12.11 ± 1.22) (Fig. 5). For the Highs, the SSS score significantly correlated with the illusory feeling of ownership measured after asynchronous stimulation in hypnosis (Spearman rho = 0.539, *p* = 0.012) (see Supplementary Information, Fig. S1) and it correlated with the proprioceptive drift measured after asynchronous stimulation in the normal waking state (Spearman rho = 0.565, *p* = 0.008) (Fig. S2). With regards to the synchronous stimulation, no statistically significant correlation was found between the SSS score and the illusory feeling of ownership (normal waking state: *p* = 0.197; hypnosis: *p* = 0.408) and between the SSS score and the proprioceptive drift (normal waking state: *p* = 0.670; hypnosis: *p* = 0.465). Finally, for the Lows no statistically significant correlation was found (all *p-values* > 0.311).

## Discussion

With this study we wanted to determine whether hypnosis induction and level of hypnotic suggestibility can alter the sense of body ownership at the RHI. To do this, we applied for the first time the RHI paradigm in hypnosis and investigated for differences in the sense of body ownership compared to normal waking state in Highs and Lows. We found a dissociated pattern between the two components of the illusion: for the illusory feeling of ownership the very same pattern could be observed for the two groups, with higher scores recorded after synchronous than after asynchronous stroking in normal waking state and in hypnosis, whereas the proprioceptive drift appeared to be differentially modulated by hypnosis and hypnotic suggestibility and was increased in the Highs and decreased in the Lows after hypnosis induction. Given that the two groups presented a different pattern at the proprioceptive component of the RHI, we can rule out a mere effect of habituation to the RHI task to explain our findings. As better discussed below, these findings highlight the interaction between hypnosis induction and an individual’s level of hypnotic suggestibility in modulating sense of body ownership.

### Hypnosis induction differently modulates the proprioceptive drift in Highs and Lows

The role of hypnotic suggestibility in modulating proprioceptive drift was demonstrated in a recent study, in which a positive correlation was found between HGSHS:A scores and proprioceptive drift^17^. The study authors suggested that high hypnotic suggestibility is associated with better attentional ability which may, in turn, facilitate the multisensory integration of visual and proprioceptive inputs necessary for proprioceptive drift^17^. A similar mechanism may be suggested for our findings after hypnotic induction. Interestingly, Walsh and colleagues^17^ made a prediction at the end of their paper: “[…] *a formal hypnotic induction procedure which can help participants “to enter a hypnotic state”*^30^ *is predicted to enhance attentional focus thereby increasing the implicit response to the RHI*”. Our findings confirm Walsh’s prediction.

Differently from our study, Walsh and colleagues^17^ measured the RHI only in the normal waking state and considered the hypnotic suggestibility score as a continuous variable against which they correlated the proprioceptive drift. In our study, we applied for the first time the RHI not only in the normal waking state but also in hypnosis. Since, the Highs and the Lows showed differences in brain activation in the normal waking state and in hypnosis^18–21^, the most suitable way to tackle potential differences at the RHI in the two mental states is via a categorical approach. Here, we used hypnotic suggestibility as a tool to categorize the study participants in Highs and Lows in order to better define different patterns at the RHI. This approach enabled us to investigate more deeply the influence of personality traits and also of different mental states in modulating the sense of body ownership.

We found that in the normal waking state, synchronous stimulation caused in both groups a drift of the subject’s own hand toward the rubber hand, whereas asynchronous stimulation caused a drift away from the rubber hand (a pattern commonly observed in the RHI). Differently from our prediction and from the findings of Walsh and colleagues^17^, we did not observe a greater proprioceptive drift in the Highs than the Lows in normal wakefulness. This may suggest that hypnotic suggestibility *per se* was not related to the perceptual aspects of the RHI in our study but rather that it interacted with hypnotic induction in inducing a change in the proprioceptive drift. In brief, after hypnosis induction, the proprioceptive drift computed as an illusion index was increased in the Highs and decreased in the Lows. Despite the reduced proprioceptive drift, the Lows preserved the illusory feeling of ownership, suggesting that they could embody the rubber hand in their own body representation^2^. This finding is consistent with the notion that an illusory feeling of ownership and proprioceptive drift constitute two distinct components of the RHI^23,24,31–33^. Support for this notion comes from studies showing that proprioceptive drift can occur independent of the illusory sense of ownership^23,32–35^ and that the perceived hand position does not affect the illusory feeling of ownership^24^.

By definition, the Highs and the Lows showed strong and weak response to hypnosis, respectively. Hence, the increase in proprioceptive drift in the Highs and the decrease in proprioceptive drift in the Lows after hypnosis induction seem to closely resemble the different ideomotor response that the two groups typically present during hypnotic induction: while the Highs were defined by their easiness of ideomotor response during hypnotic induction (i.e., “you will feel your arm falling down”), the Lows displayed a lack of ideomotor response^36–41^. The differential modulation of proprioceptive drift in the two groups could be indicative of a change of activation in brain regions associated with the recalibration of limb positioning.

We know from previous RHI studies, for instance, that an illusory feeling of ownership and proprioceptive drift are associated with activation in different brain regions^42^. While the illusory feeling of ownership is mainly associated with activation in the premotor cortex and the cerebellum^43–46^, proprioceptive drift is associated with activity in various brain regions, including the right posterior insula, the right frontal operculum, the inferior parietal lobule, and the extrastriate body area^22,42,44–48^. Of note, some of these regions overlap with a brain network, which is differently activated in Highs and Lows in hypnosis. Evidence from a functional magnetic resonance imaging (fMRI) study investigating resting state brain activation under hypnosis showed that activity in the insula, among other brain regions, is decreased in Low responders^18^. The insula has been found to positively correlate with the proprioceptive drift^22^. We may speculate, with caution, that the reduced activation in this brain region during hypnosis induction could have inhibited limb recalibration during the RHI in the Lows, thus resulting in a lack of proprioceptive drift.

Highs seem to have a more labile frontal-parietal network than Lows, making them more responsive to hypnotic induction^21^. Moreover, Highs present with reduced activity in the anterior default mode network during hypnosis^18^ and increased activity with increasing depth of hypnosis in some prefrontal regions, including the precentral gyrus (e.g., BA 44) and the middle frontal gyrus^20^. Interestingly, activity in the right frontal operculum (BA 44) and in the left middle frontal gyrus has been found to correlate positively with the proprioceptive drift^22^. These findings have not been replicated yet and therefore should be considered with caution. Nonetheless, we could speculate that the different patterns of brain activation between Highs and Lows in some core regions implicated in limb recalibration and self-attribution^49,50^ might explain the modulation of the proprioceptive drift during hypnosis.

It could be further hypothesized that in the present study the Highs and the Lows held a different attitude toward the illusion that could clearly emerge during hypnosis induction: while the Highs were prone to the illusion in both the normal waking state and hypnosis, the Lows may have developed resistance to the illusion, particularly to the proprioceptive component of the illusion, during hypnotic induction. It was recently demonstrated that subjects’ attitudes, induced through specific instructions, modulate response to the illusion^51^: when participants were asked to report where they *believed* their hand was, the proprioceptive drift was reduced compared to when they were asked to report where they *felt* their hand was. The study authors went on to observe that when the participants are asked to report their feelings, they reduced the “rational” resistance to the illusion, thus facilitating the proprioceptive drift^51^. This particular mental state is present before the illusion arises and so it influences *a priori* the response to the illusion. In a similar vein, we suggest that knowledge of the upcoming hypnotic induction may have induced a mental state in the Lows that inhibited recalibration of their own hand during synchronous and asynchronous stroking.

### Hypnotic suggestibility and the illusory feeling of ownership at the RHI

The raw scores for the Highs were overall higher than for the Lows after synchronous stimulation, suggesting a broad predisposition of the Highs to explicitly agree more than the Lows with the statement of ownership, independent of mental state (i.e., normal waking state and hypnosis). A similar trend was noted for the asynchronous (control) condition: in the normal waking state the Highs disagreed less than the Lows with the illusory statement of ownership, suggesting that the level of hypnotic suggestibility may influence high-order cognition rather than genuine perception. These findings notwithstanding, we observed a difference between synchronous and asynchronous stimulation in the normal waking state and hypnosis, independent of an individual’s level of hypnotic suggestibility. Moreover, when we computed the illusion index, we noted the very same pattern for the two groups, suggesting that the Highs and the Lows may well embody the rubber hand. This observation suggests that the level of hypnotic suggestibility impacts more on the proprioceptive drift (as discussed above) than on the illusory feeling of ownership.

Overall, the illusion index was greater in the normal waking state than in hypnosis for both groups. At first glance, this finding may indicate an attenuation of the illusion index in hypnosis. From a more close inspection of the data, however, it appears that it could be due to less disagreement in the asynchronous condition after hypnotic induction. A possible hypothesis is that hypnotic induction, which suggests relaxation, could have reduced the level of alertness^18,20^ in both groups and consequently the tendency to explicitly disagree with the statement. This remains a matter of speculation and the exact nature of this finding needs to be clarified with further ad-hoc investigation. We propose as a work hypothesis a role for the thalamus in mediating these effects, a subcortical structure involved in alertness^52,53^: the thalamus appears to be activated by asynchronous stimulation in the RHI^23^ and to be less active after hypnotic induction^18,20^.

### The role of sensory and hypnotic suggestibility

Sensory suggestibility was considered a secondary outcome in the present study; nevertheless, to our knowledge, this is the first attempt to investigate in the same individual the role of hypnotic and sensory suggestibility in response to the RHI paradigm. Hypnotic and sensory suggestibility seem to rely on different cognitive processes. Hypnotic suggestibility has been associated with focused attention and the ability to exclude distractors during hypnotic induction^17^; according to the study authors, however, sensory suggestibility requires the ability to imagine a non-existent, but suggested, sensation^16^. Our study associates for the first time these two dispositional traits by showing that high hypnotic suggestibility is related to high sensory suggestibility: higher levels of sensory suggestibility were recorded for the Highs compared to the Lows. It could be predicted, therefore, that the cognitive functions at the basis of these two traits may also interact in modulating response to the RHI. Furthermore, the sensory suggestibility scores for the Highs correlated positively with the illusory feeling of ownership in hypnosis and with proprioceptive drift in the normal waking state after asynchronous stroking, suggesting a link in the Highs between sensory suggestibility and the two components of the RHI when the illusion is not expected to occur (i.e., after asynchronous stimulation). These correlations, however, should be interpreted with caution, since the illusory feeling of ownership (in hypnosis) and proprioceptive drift (in wake) in the Highs after asynchronous stroking were close to zero on average.

A future area of focus is the interplay between these two personality traits, as it could unveil new insights into individual differences at the RHI.

### Limitation of the study

For this study we did not administer the control statements of the RHI questionnaire and we used the asynchronous condition as control. We decided not to collect data from other questionnaire statements due to a specific methodological constraint on conducting the RHI task in hypnosis. In detail, while a subject is hypnotized, he/she not only has to answer the illusion statement but also give a proprioceptive judgment in both the synchronous and the asynchronous condition. If we had administered the entire illusion questionnaire for each condition, we might have run the risk of participants exiting the hypnotic state due to an overload of cognitive self-assessment. To minimize this risk and ensure that we could test the hypnotized participants in expedited fashion, we focused the subjective assessment on a single illusion statement. This, however, may represent a limitation of the study, since we do not know whether the Highs would have agreed more also to the control statements, thus indicating a general tendency to rate the questionnaire statements higher and to be more compliant with the procedure. Furthermore, according to a study by Marotta *et al*.^12^ in which unspecific effects of sensory suggestibility on some control statements were found, we may predict that in High hypnotizable individuals the sensory suggestibility scores would correlate positively with the questionnaire ratings on the control statements. Despite this limitation due to the subjective component of the illusion, it remains clear that the proprioceptive drift was differently modulated after hypnotic induction based on the level of hypnotic suggestibility.

In our experimental procedure, the sensory suggestibility scale was administered at the end of the experiment, i.e., after the participant had experienced hypnosis. Although there is no direct evidence for a possible relationship between sensory suggestibility and hypnotic suggestibility, we cannot exclude that the hypnotic context itself might have influenced the measure of sensory suggestibility. Namely, some evidence hints at a context effect associated with the order and context of scales administration. In detail, the correlation between the Harvard Group Scale of Hypnotic Susceptibility, Form A, and the Creative Imagination Scale (CIS) varies depending on how the context is settled^54,55^. Furthermore, the magnitude of the association between the CIS and the Carleton University Responsiveness to Suggestion Scale varies as a function of the preamble (hypnosis versus imagination meta-suggestions) used to present the CIS^56^. Hence, future investigations are needed to better characterize the association between these two dispositional traits by administering the sensory suggestibility scale before the hypnotic induction and without the participants being informed that hypnosis would be then induced.

## Conclusion

This explorative study demonstrates for the first time that hypnosis induction influences an individual’s susceptibility to the RHI according to the level of hypnotic suggestibility. We found that, as a general trend, the Highs were more likely to agree with the body ownership statement than the Lows in both the normal waking state and hypnosis, although the subjective illusion index was similar for the two groups. Conversely, the proprioceptive drift toward or away from the rubber hand was different in the Highs and the Lows after hypnotic induction. This finding suggests that the disposition (in the Highs) or the resistance (in the Lows) to the hypnotic instructions may have influenced visuo-proprioceptive integration at the RHI with respect to the state of normal wakefulness. In addition to clarifying the factors modulating the sense of body ownership, these findings may be useful for future studies on body image in clinical settings.

## Supplementary information

Supplementary Information.

## Data Availability

The data generated and analysed during the current study are available from the corresponding author on reasonable request.
