# Supplementary material for: The rubber hand illusion in hypnosis provides new insights into the sense of body ownership
Source: Sci Rep. 2020 Mar 31;10:5706. doi: 10.1038/s41598-020-62745-x (PMC7109052; doi:10.1038/s41598-020-62745-x)
Supplement: Supplementary file 1 — Supplementary Information. [file 41598_2020_62745_MOESM1_ESM.docx]

**Supplementary information**

**The rubber hand illusion in hypnosis provides new insights into the sense of body ownership**

Mirta Fiorio^*^, Michele Modenese, Paola Cesari

Department of Neurosciences, Biomedicine and Movement Sciences, University of Verona, Verona, Italy

*[mirta.fiorio@univr.it](mailto:mirta.fiorio@univr.it)


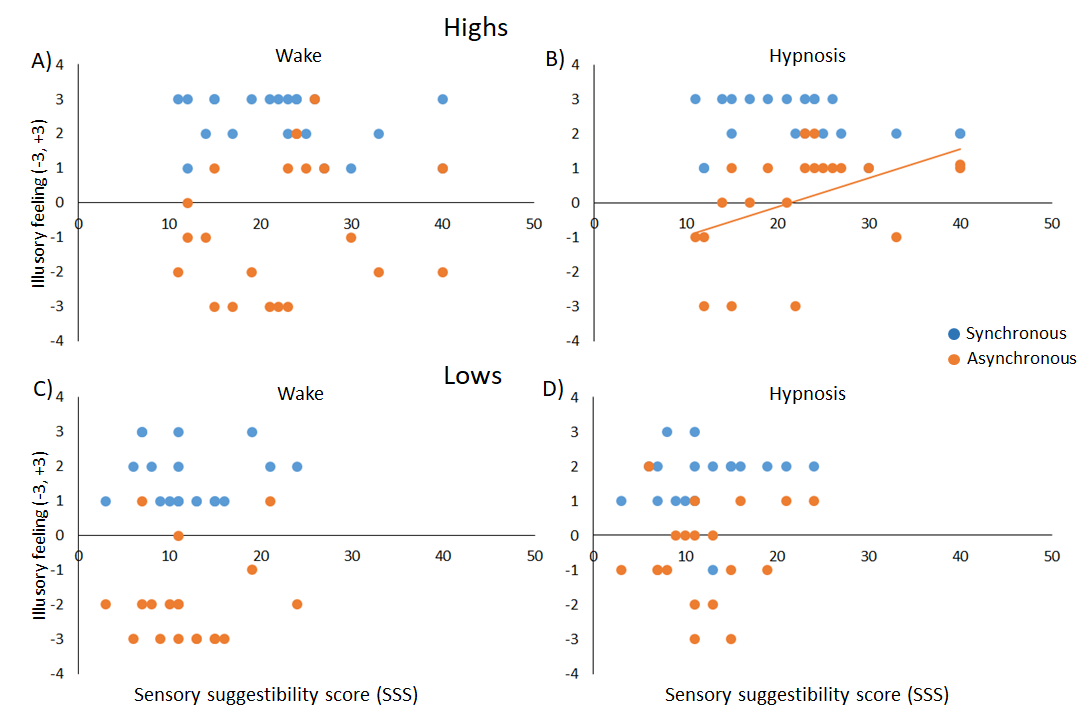


**Figure S1.** Spearman’s correlation between the illusory feeling of ownership and the total SSS score in the synchronous (blue circles) and asynchronous (orange circles) conditions in Highs (upper panels) and Lows (lower panels) in the normal waking state (A and C) and in hypnosis (B and D). A positive correlation was found in Highs between the SSS scores and the illusory feeling of ownership after asynchronous stroking in hypnosis.


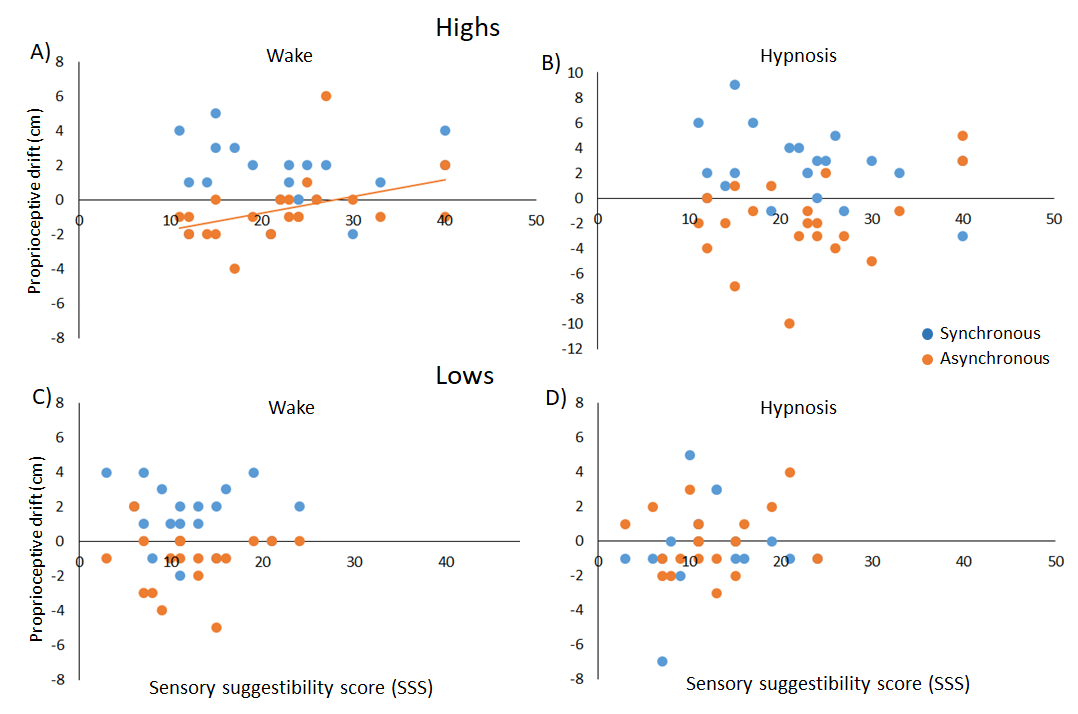


**Figure S2.** Spearman’s correlation between proprioceptive drift and the total SSS in the synchronous (blue circles) and asynchronous (orange circles) conditions in Highs (upper panels) and Lows (lower panels) in the normal waking state (A and C) and in hypnosis (B and D). A positive correlation was found in Highs between the SSS scores and the proprioceptive drift after asynchronous stroking in the normal waking state.
